# Time Trends and Treatment Pathways in Prescribing Individual Oral Anticoagulants in Patients with Nonvalvular Atrial Fibrillation: An Observational Study of More than Three Million Patients from Europe and the United States

**DOI:** 10.1155/2022/6707985

**Published:** 2022-01-31

**Authors:** Pareen Vora, Henry Morgan Stewart, Beth Russell, Alex Asiimwe, Gunnar Brobert

**Affiliations:** ^1^Epidemiology, Bayer AG, Berlin, Germany; ^2^IQVIA, Brighton, UK; ^3^Epidemiology, Bayer AB, Solna, Sweden

## Abstract

**Background:**

Data directly comparing trends in the use of different oral anticoagulants (OACs) among patients with atrial fibrillation (AF) from different countries are limited. We addressed this using a large-scale network cohort study in the United States (US), Belgium, France, Germany, and the United Kingdom (UK).

**Methods:**

We used nine databases (claims or electronic health records) that had been converted into the Observational Medical Outcomes Partnership Common Data Model with analysis performed using open-source analytical tools. We identified adults with AF and a first OAC prescription, either vitamin K antagonist (VKA) or direct oral anticoagulant (DOAC), from 2010 to 2017. We described time trends in use, continuation, and switching.

**Results:**

In 2010, 87.5%–99.8% of patients started on a VKA. By 2017, the majority started on a DOAC: 87.0% (US), 88.3% (Belgium), 93.1% (France), 88.4% (Germany), and 86.1%–86.7% (UK). In the UK, DOACs became the most common starting OAC in 2015, 2-3 years later than elsewhere. Apixaban was the most common starting OAC by 2017, 50.2%–57.8% (US), 31.4% (Belgium), 45.9% (France), 39.5% (Germany), and 49.8%–50.5% (UK), followed by rivaroxaban, 24.8%–32.5% (US), 25.7% (Belgium), 38.4% (France), 24.9% (Germany), and 30.2%–31.2% (UK). Long-term treatment was less common in the US than in Europe, especially the UK. A minority of patients switched from their index OAC in the short and long term.

**Conclusions:**

From 2010 to 2017, VKA use had significantly declined and DOAC use had significantly increased in the US and Europe. Apixaban was the most prescribed OAC in 2017, followed by rivaroxaban.

## 1. Introduction 

Patients with atrial fibrillation (AF) at increased risk of stroke require long-term treatment with oral anticoagulants (OACs) to reduce their stroke risk. The introduction of direct oral anticoagulants (DOACs) as an alternative to vitamin K antagonists (VKAs) for stroke prevention in patients with AF over the last decade has resulted in a clear shift towards greater use of these drugs in this patient population. This newer class of drugs has demonstrated at least equivalent efficacy and safety to warfarin with a lower risk of intracranial bleeding in randomized controlled trials. [1–4] Currently, four DOACs are available on the market, approved at different times for stroke prevention in AF in the last decade—dabigatran (a thrombin inhibitor) was introduced in 2010, followed by the factor Xa inhibitors rivaroxaban, apixaban and, more recently, edoxaban.

The change in the clinical landscape of OAC use away from VKAs towards DOACs and between individual DOACs, which have slightly different clinical profiles and dosing frequency, is evident from studies across several countries [5–12]. Several studies have investigated the usage patterns and switching through different methodologies and time periods, but to enable true international comparisons, a more systematic analytical approach is required. Furthermore, time trends in DOAC prescription have often been analysed as a class, and there are limited data comparing temporal trends in the use of individual DOACs among populations with AF from countries with different healthcare systems or long-term temporal patterns in the sequence of individual OAC treatments. Differences in any of these aspects of OAC treatment between individual countries could potentially reveal variations in the attitudes of physicians/patients towards different OAC treatments in different nations. This, in turn, could drive further investigation into the reasons behind these differences, with the aim of understanding how best to optimise OAC uptake in patients with AF and minimise discontinuation, which increases the risk of stroke [13–17]. Using a large-scale network study approach, we aimed to characterize and compare time trends in the prescribing VKA and individual DOACs and OAC treatment pathways (including switching), among patients with AF in routine clinical practice in Belgium, France, Germany, the United States (US), and the United Kingdom (UK).

## 2. Methods

### 2.1. Study Design and Data Sources

We conducted a retrospective cohort study using nine databases–either electronic health records (EHRs) or administrative claims–from five countries: four from US; two from the UK; and one each from Belgium, France, and Germany (Table 1). Data were available from 2010 to 2017, for all sources except for the Longitudinal Prescription Diagnosis Database in the US, which at the time of the study held information from 2011 to 2017, and the French Disease Analyzer database, which held data from 2012 to 2017. These databases had been converted to a standardised format using Observational Medical Outcomes Partnership (OMOP) common data model, [18] which was developed through public-private partnership in the US. The common data model is updated by the Observational Health Data Sciences and Informatics (OHDSI) community—an interdisciplinary collaboration based on the principle of open-source data analytics [19]. Details about the common data mode can be found elsewhere; [18] however, briefly, the common data model enables different databases, with their specific coding system, to be analysed in a standardised way.

### 2.2. Study Cohorts

We included patients aged at least 18 years with a diagnosis of AF (see Supplementary Table 1) and a first prescription or dispensing for OAC, either a VKA or a DOAC (dabigatran, rivaroxaban, apixaban, or edoxaban) during the study period (see Supplementary Table 2 for Anatomical Therapeutic Chemical Classification codes). The start of the study was 1 January 2010 for all data sources except for the US Longitudinal Prescription Diagnosis database (US LPD) and the French Disease Analyzer database, where the start date was 1 January 2011 and 1 January 2012, respectively, due to the small number of patients starting OAC treatment in 2010 (7 patients in US LPD and 23 patients in the French Disease Analyzer database). The end of the study period was the date of the latest available data in 2017 for each database. Patients were required to have a minimum of 1 year of observation before the start of the study with no prescription for any OAC during this time. The index date for each patient was the date of the first OAC prescription (index prescription) during the study period. For each patient, we extracted information on age at the index date and sex.

### 2.3. Assessment of Treatment Patterns

For each calendar year in the study, we performed the following steps. Firstly, we identified the first OAC prescribed to each patient newly started on OAC therapy. Secondly, we described long-term treatment pathways for patients whose index prescription was during 2010–2016 and who were still available for observation in 2017. We identified whether they were prescribed an OAC at any point during 2017, irrespective of any treatment gaps. If so, whether this was for the same or a different OAC (i.e., they had switched or discontinued their initial therapy by 2017). Thirdly, we followed up all patients from their index prescription for a maximum of two years to determine treatment continuation and switching patterns during this two-year observation period (including whether they had switched more than once, either to a second different OAC or back to the index OAC). In this analysis, only patients still available for observation at 2 years after their index prescription were included.

### 2.4. Data Analysis

The age of the study population was presented as the mean with standard deviation, and the sex distribution was presented using the frequency count and percentage. For each calendar year, we calculated the percentage of patients in each AF study cohort initiated on a specific OAC medication. Bar charts and sunburst plots were produced to visualise treatment pathways over the study years. All analyses were performed using the R study package based on R studio across.

## 3. Results

A description of each AF study cohort is shown in Table 2. Mean age at first OAC prescription ranged from 56.2 years (SD 7.1; US CCAE database) to 78.0 years (SD 7.3; US MDCR database), and females accounted for between 31% (UK CCAE database) and 47% (US MDCR and Germany DA database).

### 3.1. Temporal Trends in VKA and DOAC Initiation

**Figure 1:**
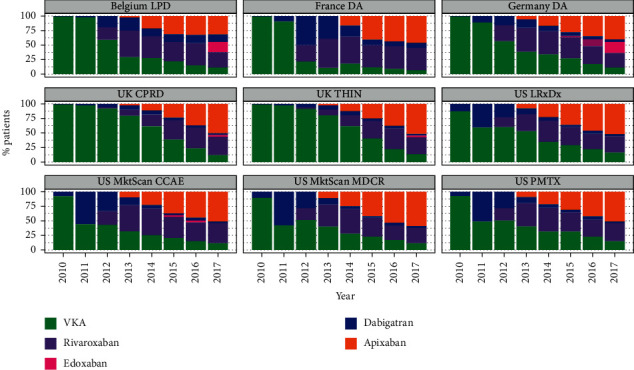
The frequency distribution of each index OAC for each database across the study period (patients with AF).

The frequency distribution of each index OAC for each database across the study period is shown in Figure 1. Over the study period, there was a clear decline in the percentage of patients with AF initiated on a VKA with a corresponding increase in the proportion initiated on a DOAC, which was seen in all five countries. In 2010, between 87.5% and 99.8% of patients prescribed an OAC for AF were initiated on a VKA (US, 87.5%–93.1%; Belgium, 99.4%; France, 98.6%; Germany, 98.9%; the UK, 99.8%). By 2017, the majority were initiated on a DOAC (US, 83.7%–87.0%; Belgium, 88.3%; France, 93.1%; Germany, 88.4%; the UK, 86.1%–86.7%). The uptake of DOACs was the slowest in the UK. In 2013, 19.4% (IMRD-UK) and 19.5% (CPRD-GOLD) of patients with AF were initiated on DOACs compared with Belgium, 69.8%; France, 88.6%; Germany, 61.5%; and the US, 59.3%–67.2%. In the UK, DOACs overtook VKAs as the most common starting OAC in 2015; this was 2-3 years later than the other countries.

By 2017, apixaban was the most common starting OAC in all five countries (US, 50.2%–57.8%; Belgium, 31.4%; France, 45.9%; Germany, 39.5%; the UK; 49.8%–50.5%), followed by rivaroxaban (US, 24.8%–32.5%; Belgium, 25.7%; France, 38.4%; Germany, 24.9%; the UK, 30.2%–31.2%). The use of dabigatran was mainly seen during 2011–2012 in the US and from 2012 to 2013 in Belgium, France, and Germany, with minimal use seen in the UK. Edoxaban—the newest DOAC on the market—was the starting OAC in 2017 in 18.8% of patients (*n* = 1700) with AF in Germany and 18.5% (*n* = 900) in Belgium; few patients with AF (≤2.5%) in France, the UK, and the US were initiated on edoxaban in 2017.

### 3.2. Long-Term OAC Discontinuation

**Figure 2:**
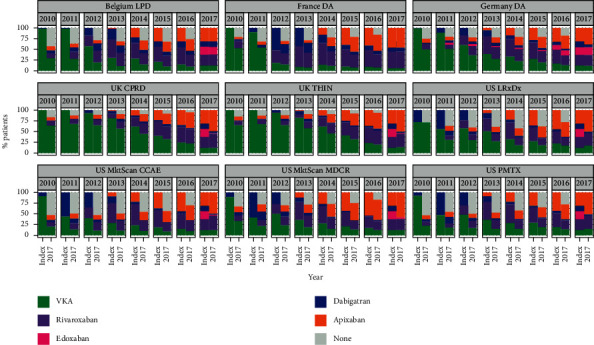
Frequency distribution of each index OAC for each calendar year and first OAC prescribed in 2017 (patients with AF still available for observation in 2017).

The frequency distribution of the index OAC (for individual calendar years 2010–2016) and the first OAC prescribed in 2017, for the subgroup of patients still available for observation in 2017, is shown in Figure 2 and Supplementary Table 3. Of the patients initiating OAC treatment in 2010 (or 2011 for the US LPD and French Disease Analyzer database), a notable proportion had discontinued OAC therapy by 2017 (US, 32.9%–54.2%; Belgium, 42.9%; France, 33.3%; Germany, 25.6%; the UK, 15.4%–16.1%). Irrespective of the year that OAC treatment started, long-term treatment was not as common in the US as in the European countries, especially the UK. Also, irrespective of the year that OAC treatment started, the majority of patients initiated on a VKA in the UK remained on a VKA in 2017. Similarly, the majority of patients initiated on a specific DOAC remained on that DOAC. This pattern for long-term continuation of VKAs and the same DOAC was also seen in France and Germany but was less evident for VKAs, at least in Belgium and the US.

### 3.3. Two-Year Switching Patterns

**Figure 3:**
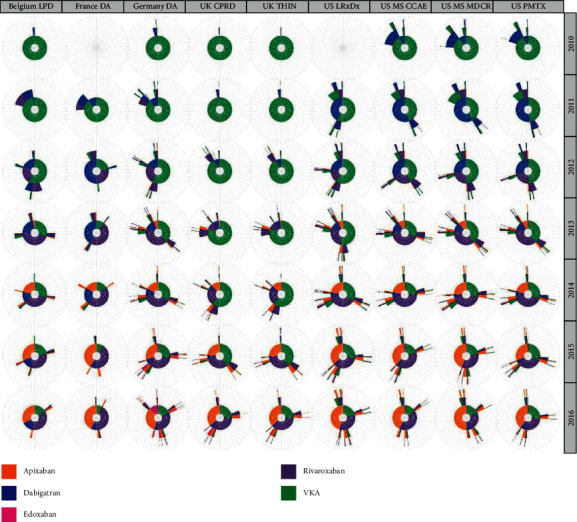
Two-year OAC treatment sequences (irrespective of gaps in treatment) in patients with AF by database and study year (patients still available for observation at 2 years after their index prescription). Note: the inner circle of each sunburst plot shows the percentage of patients prescribed each OAC type (first OAC prescription) in that year, coloured segments in the next outer circle show the second OAC prescribed (if any) at any time during the two-year follow-up period (i.e., the first OAC switch), and coloured segments in the second outer circle (if any) show the third OAC prescribed (if any) at any time during the two-year follow-up period (either a switch back to the original OAC prescribed or a switch to another different OAC. Also, very few patients were available for analysis in the US Longitudinal Prescription Diagnosis database and the French Disease Analyzer database in 2010. Hence, results are not shown for these databases in this year. Each cell in the sunburst plot represents 2%.

Switching patterns within a maximum of two-year follow-up by database and index OAC calendar year, depicting the first and potentially second and third line of treatment, are illustrated in Figure 3. Across countries and calendar years, most patients remained on the same OAC during a maximum of two years after initiating therapy, especially those starting on apixaban or rivaroxaban. For example, in Germany, among patients starting OAC therapy in 2016, 2-year same-OAC proportions were 90.6% for apixaban, 69.5% for dabigatran, 79.3% for rivaroxaban, and 69.9% for VKAs. In the US in 2014 (using the IQVIA Pharmetrics database), 2-year same-OAC rates were 89.8% for apixaban, 65.5% for dabigatran, 83.0% for rivaroxaban, and 78.5% for VKAs. Accordingly, in each country, a much smaller proportion switched treatments, mostly from a VKA to a DOAC for the first time, or from their starting DOAC to another DOAC. Among patients initiating dabigatran in the US in 2011, a notable proportion switched to a VKA within the following two years: 31.9% in the Marketscan MDCR database, 16.5% in the Marketscan CCAE database, 31.9% in the US LPD, and 22.5% in the IQVIA Pharmetrics database. Only a very small proportion of patients had two switches in treatment during this time period, and this was often back to the index OAC.

## 4. Discussion

This population-based observational study, which analysed data from nine data sources across four European countries and the US, provides insights into the prescribing of different OACs for stroke prevention in patients with AF across multiple healthcare systems during the last decade. Using a harmonised methodology, we were able to compare long-term temporal trends between countries with different healthcare systems and identify differences in the uptake of individual DOACs, continuation rates, and switching. Across countries, we found a clear shift away from VKAs and towards DOACs as the most common starting OAC therapy prescribed for stroke prevention in the AF patient population. Long-term continuation with OAC therapy was the highest in the UK and lowest in the US. Most patients remained on their starting OAC in the short and long term, with only a small proportion switching. Additionally, our study has demonstrated the application of a new analytic tool in the OHDSI toolbox to inform about its existence and encourage its use by others.

Our observed declines in VKA use and increases in DOAC use are consistent with several other studies on this topic from Europe [6, 7, 20–22] and the US [9, 23]. This may partly indicate the increasing confidence of physicians in prescribing DOACs to patients with AF in clinical practice. In addition to the favourable benefit-risk profile of DOACs over VKAs, their more predictable pharmacokinetics avoids the need for regular monitoring of patients' international normalised ratio (INR) that is needed with VKA. Furthermore, the trends in the uptake of individual DOACs reflect their different approval times in the US and European markets. For example, the earlier approval of dabigatran in the US (October 2010) than Europe (August 2011) is reflected in the much higher proportion of dabigatran use in the US cohorts in our study in 2011. Similarly, the approvals of the different DOACs at different times (starting with dabigatran, then rivaroxaban, apixaban, and edoxaban) are reflected in the gradual uptake of these individual drugs during progressive study years. It is also possible that the 2012 European Society of Cardiology (ESC) guidelines [24], which recommended DOACs as a treatment option in the context of stroke prevention in AF, boosted uptake shortly after in France, Germany, and Belgium; however, reasons for the slower uptake in the UK are unclear. In the UK, the relevant National Institute of Health and Care Excellence recommendations were published in March 2012 (dabigatran), [25] May 2012 (rivaroxaban) [26], and February 2013 (apixaban), [27], which were only 7-8 months after the respective EMA approvals (August 2011, September 2011, and September 2012). They are therefore unlikely to be the sole reason for the slower uptake of DOACs in the UK. Other explanations are speculative but could be related to a higher degree of scepticism by UK physicians due to several possible reasons, including the noninferiority of DOACs to warfarin, their higher cost, and a lack of established protocols for dealing with bleeding. Additionally, in the UK, DOACs were not initially included in formularies and were considered a second-line therapy.

Clinical guidelines recommend that patients with NVAF at high risk of stroke continue with lifelong OAC therapy in order to gain the thromboembolic protection they need and to minimise stroke risk [28, 29]. A recent study by Garcia Rodriguez et al. [13] showed that patients with NVAF who discontinue OAC therapy have a significant two-to-three-fold higher risk of ischaemic stroke compared with those who continue therapy, consistent with previous smaller studies on this topic [14–17]. Our present study suggests substantial regional variation in levels of OAC discontinuation, and the notable difference between the US and the UK is consistent with previous reports [30, 31]. It is possible that the higher OAC discontinuation rates seen in the US cohorts were due to their greater proportion of males/younger demographic. The highest discontinuation rates were seen in the two US cohorts that had both the highest proportion of males and the lowest mean age: US Marketscan CCAE database (53.1% discontinuation, 69% male, mean age 56.2 years) and the US PMTX database (54.2% discontinuation, 63% male, mean age 63.1 years). Also, the lowest discontinuation rate among the four US cohorts was seen in the Marketscan MDCR database (32.9%, which includes only individuals aged ≥65 years, mean age in the study cohort 78.0 years). However, adjusted analyses would be needed to see whether younger age and male gender were independent driving factors for OAC discontinuation, and this was beyond the scope of the study. The low proportion of patients switching OAC medication in our study is also in line with the low rates of switching seen in other studies [14, 32, 33]. The notable switching to a VKA among patients started on dabigatran in 2011 most likely reflects concerns over bleeding risk with dabigatran use that arose around this time [34], which led to further evaluation and was later refuted [35, 36].

A key strength of our study was the use of multiple large population-based datasets that were standardised using OMOP CDM from countries with different healthcare systems, which were evaluated using the same analytical code. This enabled an overarching understanding of the clinical landscape of OAC treatment for AF since 2010. We provided a clear graphical overview of a vast quantity of data from several countries during a specific time period, facilitating the interpretation of temporal trends and intercountry comparisons. Other study strengths include the large size of the study cohorts, the long follow-up duration for many patients, and the analysis of all DOACs currently available to prescribers. We were also confident that a prescription for a different OAC after the index OAC represented a switch in drugs because OACs are never prescribed in combination. The EHR databases included in the study are considered representative of the wider respective population from which the dataset sample was drawn, and therefore, findings from these datasets can be considered to have good external validity. However, findings from the claimed databases are limited to the wider insured populations from which the samples were drawn. Another limitation of the study is that while the sunburst plots provide information on OAC switching, they do not indicate the exact switching date. Sample sizes were at least a magnitude smaller for Belgium and France, and therefore, the findings may not have been as accurate as those from the larger datasets from the US, UK, and Germany.

In conclusion, between 2010 and 2017, the clinical landscape of OAC use for stroke prevention in patients with AF changed significantly across the US, UK, and Europe, with significant declines in VKA use and corresponding increases in DOAC use. By 2017, apixaban was the most prescribed OAC in the US, Germany, France, Belgium, and the UK, followed by rivaroxaban. Further monitoring of OAC prescribing trends in more recent and future years would be beneficial for the continued evaluation of OAC prescribing trends in the context of stroke prevention in AF.

## Figures and Tables

**Table 1:** Description of databases used in the study.

Database	Data type	Country	Years covered^*∗*^	Description
IQVIA Belgium Longitudinal Patient Database (LPD)	EHR	Belgium	2010–2017	(i) Data coverage of ∼2 million patients, 688 care sites, 15 million visits, and 140 million service records.
				(ii) Dates of service include 2008 to present.

IQVIA France Disease Analyzer (DA)	EHR	France	2012–2017	(i) Data collected from outpatient, general practitioner practices, and medical centers for all ages. Data coverage includes more than 10.9 million patients, 3,100 providers, 550 care, sites over 458.2 million medical events and services.
				(ii) Dates of service include from 1997 to present.

IQVIA Germany Disease Analyzer (DA)	EHR	Germany	2010–2017	(i) Data from physician practices and medical centers for all ages; mostly primary care physician data; however, some data from specialty practices (where practices are electronically connected to each other) and some laboratory data are included.
				(ii) Dates of service include from 1992 to present.

IQVIA Medical Research Database (IMRD)	EHR	UK	2010–2017	(i) Primary care data contributed from practices across the UK.
				(ii) Data coverage includes 15 million patients, 5 million providers, 793 care sites, and more than 5 billion service records.
				(iii) Dates of service include from 1989 to present.

CPRD-GOLD	EHR	UK	2010–2017	(i) Primary care data contributed from practices across the UK.
				(ii) Data coverage includes over 11.3 million patients from 674 practices with 4.4 million active (alive, currently registered) patients meeting quality criteria.
				(iii) Dates of service include from 1987 to present.

IQVIA Open Claims (LRxDx)	Claims	US	2011–2017	(i) Claims at the anonymized patient level collected from office-based physicians and specialists via office management software and clearinghouse switch sources for the purpose of reimbursement. A subset of medical claims data have adjudicated claims.
				(ii) Covers the total US population (unadjudicated claims from multiple data sources)
				(iii) Covers claims from 2010 to present.

IQVIA Pharmetrics Plus (PMTX+)	Claims	US	2010–2017	(i) Closed claims database of fully adjudicated pharmacy, hospital, and medical claims at the anonymized patient level sourced from commercial payers.
				(ii) Covers claims from 2006 to present.

Marketscan CCAE	Claims	US	2010–2017	(i) Insurance claims information for privately employer-insured individuals.
				(ii) Generally includes data from active employees, Comprehensive Omnibus Budget Reconciliation Act (COBRA) continues, early (nonmedicare) retirees, and dependents who are younger than 65 years of age.
				(iii) In 2016, the database held 43.6 million person-years of data.

Marketscan MDCR				(i) Claims data on medicare-eligible active and retired employees and their medicare-eligible dependents from employer-sponsored supplemental plans (predominantly fee-for-service plans) aged 65 years or over. Only plans where both the medicare-paid amounts and the employer-paid amounts were available and evident on the claims were selected for this database.
				(ii) As of 19 October 2018, MDCR contained 9.89 million patients.
				(iii) Patient-level observations from January 2002 through December 2016.

^*∗*^At the time the study was carried out. CCAE: Commercial Claims and Encounters; CPRD: Clinical Practice Research Datalink; DA: Disease Analyzer; EHR: electronic health records; IMRD: IQVIA Medical Research Data UK; LPD: Longitudinal Patient Database; LRxDx: Longitudinal Prescription Diagnosis database; MDCR: Medicare Supplemental and Coordination of Benefits; PMTX: Pharmetrics; SD: standard deviation.

**Table 2:** Basic description of the AF study cohorts.

Data source	Patients (N)^*∗*^	Mean age (±SD) at first OAC prescription	% female
Belgium LPD	6546	74.5 (10.5)	45
France DA	5053	73.6 (10.5)	43
Germany DA	72,297	74.1 (10.1)	47
UK THIN	52,720	74.1(10.5)	44
UK CPRD	48,830	74.3 (10.5)	44
US LRxDx	3,195,578	70.3 (10.5)	45
US PMTX	193,118	63.1 (11.0)	35
US Marketscan CCAE	97,220	56.2 (7.1)	31
US Marketscan MDCR	170,971	78.0 (7.3)	47

^*∗*^Some patients could potentially contribute to more than one database, for example, THIN and CPRD databases in the UK. AF: atrial fibrillation; CCAE: Commercial Claims and Encounters; CPRD: Clinical Practice Research Datalink; DA: Disease Analyzer; IMRD: IQVIA Medical Research Data UK; LPD: Longitudinal Patient Database; LRxDx: Longitudinal Prescription Diagnosis database; MDCR: Medicare Supplemental and Coordination of Benefits; OAC: oral anticoagulant; PMTX: Pharmetrics; SD: standard deviation.

## Data Availability

Data are available from the corresponding author upon reasonable request.

## Abbreviations

AF: Atrial fibrillation
CCAE: Commercial claims and encounters
CPRD: Clinical Practice Research Datalink
DA: Disease Analyzer
LPD: Longitudinal Patient Database
LRxDx: Longitudinal Prescription Diagnosis database
MDCR: Medicare Supplemental and Coordination of Benefits
OAC: Oral anticoagulant
PMTX: Pharmetrics
THIN: The Health Improvement Network.

## Additional Points

What is already known about this topic? (i) Several country-specific studies have described time-trends in oral anticoagulant (OAC) use for atrial fibrillation (AF). (ii) True international comparisons require a systematic analytical approach. What does this article add? (i) In a large-scale network cohort study of OAC therapy for AF (2010–2017) in Belgium, France, Germany, the United States (US), and the United Kingdom (UK), substantial regional variation was seen for OAC discontinuation, being notably highest in the UK and lowest in the US.

## Supplementary Materials

Supplementary Materials
*Supplementary Table 1*. Codes to identify AF. *Supplementary Table 2*. Codes for antithrombotic agents used for stroke prevention in patients with AF. *Supplementary Table 3*. Proportion of patients who discontinued OAC therapy by 2017, according to the year of the index OAC prescription (2010–2016).
