# Supplementary material for: Time Trends and Treatment Pathways in Prescribing Individual Oral Anticoagulants in Patients with Nonvalvular Atrial Fibrillation: An Observational Study of More than Three Million Patients from Europe and the United States
Source: Int J Clin Pract. 2022 Jan 31;2022:6707985. doi: 10.1155/2022/6707985 (PMC9159118; doi:10.1155/2022/6707985)
Supplement: Supplementary Materials — Supplementary Table 1. Codes to identify AF. Supplementary Table 2. Codes for antithrombotic agents used for stroke prevention in patients with AF. Supplementary Table 3. Proportion of patients who discontinued OAC therapy by 2017, according to the year of the index OAC prescription (2010–2016). [file 6707985.f1.pdf]

Supplementary Table 1. Codes to identify AF.

**Target diagnosis (SNOMED)**	**Included SNOMED codes and descriptions**
Atrial fibrillation	49436004, 195080001, 233910005, 233911009, 282825002, 300996004, 314208002, 426749004, 440028005, 440059007, 706923002, 715395008, 120041000119109<br><br>AF and flutter, Controlled AF, Lone AF, Non-rheumatic AF, Chronic AF, Paroxysmal AF, Rapid AF, Permanent AF, Persistent AF, Familial AF, AF with rapid ventricular response, Longstanding persistent AF

AF, atrial fibrillation

Supplementary Table 2. Codes for Antithrombotic agents used for stroke prevention in patients with AF.

**Classification**	**ATC code (4th class)**	**ATC code (5th class)**
vitamin K antagonist	B01AA	dicoumarol, phenindione, warfarin, phenprocoumon, acenocoumarol, ethyl biscoumacetate, clorindione, diphenadione, tioclomarol, fluindione
Direct thrombin inhibitors	B01AE	dabigatran etexilate
Direct factor Xa inhibitors	B01AF	rivaroxaban, apixaban, edoxaban

AF, atrial fibrillation; ATC, Anatomical Therapeutic Chemical

Supplementary Table 3. Proportion of patients who discontinued with OAC therapy by 2017, according to year of the index OAC prescription (2010–2016).
Note: this analysis included only patients still available for observation in 2017.

**Database**	**Year of index OAC prescription**	**N**	**% discontinued by 2017**
US LRxDx	2010	7	28.6
	2011	248,497	37.5
	2012	260,636	40.4
	2013	374,200	39.0
	2014	372,428	41.6
	2015	412,452	38.6
	2016	470,267	28.4
US MarketScan CCAE	2010	3321	53.1
	2011	6450	48.6
	2012	9542	52.5
	2013	12,661	49.0
	2014	15,328	46.4
	2015	18,848	42.1
	2016	24,793	29.7
US MarketScan MDCR	2010	2110	33.0
	2011	4359	27.7
	2012	6526	31.2
	2013	8581	28.2
	2014	9998	26.1
	2015	11,652	24.6
	2016	14,775	16.3
US PMTX	2010	3046	54.2
	2011	4989	46.2
	2012	5544	49.4
	2013	7888	46.9
	2014	10,655	42.2
	2015	17,398	35.1
	2016	20,572	26.9
UK CPRD	2010	1236	16.2
	2011	1501	13.1
	2012	2094	12.0
	2013	2538	9.7
	2014	3431	9.0
	2015	4426	7.4
	2016	4931	5.8

Database	Year of index OAC prescription	N	% discontinued by 2017
UK THIN	2010	1402	15.5
	2011	1752	13.4
	2012	2516	12.6
	2013	3180	10.0
	2014	4191	9.5
	2015	5368	7.7
	2016	6021	5.6
Germany DA	2010	1730	25.6
	2011	2987	20.8
	2012	4722	22.4
	2013	6342	22.2
	2014	7480	22.0
	2015	8530	20.6
	2016	9989	18.7
Belgium LPD	2010	198	42.9
	2011	293	37.2
	2012	428	29.7
	2013	585	32.0
	2014	581	29.9
	2015	694	32.4
	2016	813	25.8
France DA	2010	23	21.7
	2011	39	33.3
	2012	255	31.4
	2013	390	28.7
	2014	377	23.1
	2015	607	20.6
	2016	888	16.4

CCAE, Commercial Claims and Encounters; CPRD, Clinical Practice Research Datalink; DA, Disease Analyzer; LPD, Longitudinal Patient Database; LRxDx, Longitudinal Prescription Diagnosis database; MDCR, Medicare Supplemental and Coordination of Benefits; OAC, oral anticoagulant; PMTX, Pharmetrics; THIN, The Health Improvement Network; US, United States; UK, United Kingdom
